# Deep mRNA Sequencing Analysis to Capture the Transcriptome Landscape of Zebrafish Embryos and Larvae

**DOI:** 10.1371/journal.pone.0064058

**Published:** 2013-05-20

**Authors:** Hongxing Yang, Yan Zhou, Jianlei Gu, Shuying Xie, Yao Xu, Genfeng Zhu, Lei Wang, Jiyue Huang, Hong Ma, Jihua Yao

**Affiliations:** 1 State Key Laboratory of Genetic Engineering, Institute of Plant Biology, Center of Evolutionary Biology, School of Life Science, Fudan University, Shanghai, China; 2 State Key Laboratory of Genetic Engineering, Department of Microbiology, School of Life Sciences, Fudan University, Shanghai, China; 3 State Key Laboratory of Genetic Engineering, Institute of Genetics, School of Life Sciences, Fudan University, Shanghai, China; 4 Institute of Biomedical Sciences, Fudan University, Shanghai, China; IGBMC/ICS, France

## Abstract

Transcriptome analysis is a powerful tool to obtain large amount genome-scale gene expression profiles. Despite its extensive usage to diverse biological problems in the last decade, transcriptomic researches approaching the zebrafish embryonic development have been very limited. Several recent studies have made great progress in this direction, yet the large gap still exists, especially regarding to the transcriptome dynamics of embryonic stages from early gastrulation onwards. Here, we present a comprehensive analysis about the transcriptomes of 9 different stages covering 7 major periods (cleavage, blastula, gastrula, segmentation, pharyngula, hatching and early larval stage) in zebrafish development, by recruiting the RNA-sequencing technology. We detected the expression for at least 24,065 genes in at least one of the 9 stages. We identified 16,130 genes that were significantly differentially expressed between stages and were subsequently classified into six clusters. Each revealed gene cluster had distinct expression patterns and characteristic functional pathways, providing a framework for the understanding of the developmental transcriptome dynamics. Over 4000 genes were identified as preferentially expressed in one of the stages, which could be of high relevance to stage-specific developmental and molecular events. Among the 68 transcription factor families active during development, most had enhanced average expression levels and thus might be crucial for embryogenesis, whereas the inactivation of the other families was likely required by the activation of the zygotic genome. We discussed our RNA-seq data together with previous findings about the Wnt signaling pathway and some other genes with known functions, to show how our data could be used to advance our understanding about these developmental functional elements. Our study provides ample information for further study about the molecular and cellular mechanisms underlying vertebrate development.

## Introduction

The functional and physiological status of a cell, tissue or organism depends on its transcriptome, the complete set of transcripts. The analysis of (nearly) all transcripts by transcriptome profiling has proved a powerful tool for revealing the gene activities underlying various biological processes, including developmental programs and genetic disorders. Vertebrate embryogenesis represents one of the most complicated developmental programs marked by a series of critical events including cleavage, blastulation, gastrulation, and somitogenesis, which are directed by dramatic reprogramming of the genome-wide transcription activities and regulatory networks [1], [2]. Transcriptome analysis is thus of crucial importance to understand the molecular basis of embryogenesis and the causes of developmental defects [3], [4].

During the past decade, the transcriptomes of vertebrate embryogenesis have been studied using different platforms including microarrays and serial analysis of gene expression [4], [5], for human [1], [6], and model organisms such as mouse [2], [7], [8] and zebrafish [9], [10]. Furthermore, high-throughput RNA in situ hybridization has also been employed to obtain an anatomical atlas of embryonic transcriptomes in mouse [11]. These studies have greatly advanced our understanding about the dynamic nature of the global expression patterns during vertebrate embryogenesis. Recently, the RNA-sequencing (RNA-seq) technology has emerged as a very powerful tool for transcriptomic studies [12]. For example, RNA-seq has been used to analyze the transcriptomes of mouse embryonic and neonatal cortices [13] and of a single mammalian cell, such as an oocyte or an embryonic blastomere [14]. Compared to the microarray techniques, RNA-seq can profile the abundance of RNA transcripts in greater depth and accuracy, can detect alternative splicing variants and novel transcripts more effectively, and does not require an assembled genome sequence [15]. Consequently, it has rapidly become the state-of-the-art platform for transcriptomic studies.

Zebrafish is an excellent model organism for understanding vertebrate development. Its advantages include a short generation time, large clutch size, and transparent embryos that develop externally and rapidly; these features facilitate genetic manipulation and convenient observation of embryonic development [16], [17]. More than 4000 zebrafish mutants have been obtained and the phenotypes of some of the mutants were similar to defects associated with related human diseases [10], [18]. Moreover, genetic tools such as transgenesis and morpholino knockdown assays for zebrafish were well developed and highly effective. Consequently, zebrafish as also serves as an excellent model system to study vertebrate developmental disorders and human diseases [9], [18].

Currently, the sequencing of the zebrafish genome has not been completed and the annotation is still ongoing. Genome-wide measurements of gene expression could provide additional evidence for the gene model annotations, while at the same time help elucidating how gene activities are coordinated to form a genetic network regulating zebrafish embryogenesis. RNA-seq has already been employed to monitor the transcriptome responses to mycobacterium infection and the transcriptome dynamics of some stages of embryonic development in zebrafish [19]–[21]. Also, a large cohort of long non-coding transcripts active during embryogenesis has been discovered using RNA-seq [22]. However, large gaps still remain in our understanding about the comprehensive transcriptome dynamics during the early as well as the late stages of embryonic development in zebrafish.

In this study, we utilized the SOLiD™ 3 (Life Technologies) platform to analyze the transcriptomes of 9 different developmental time points covering 7 different developmental periods: cleavage, blastula, gastrula, segmentation, pharyngula, hatching and early larva. From these transcriptomes, we identified over 4000 genes that were preferentially expressed in one of the developmental stages, revealing enrichment for stage-specific biological pathways. We were able to classify the large cohort of significantly differentially expressed genes between stages into six clusters, each with distinct expression patterns, providing a framework for understanding the transcriptome dynamics during embryonic development. In particular, distinct expression patterns were also uncovered among and within transcription factor families, suggesting their importance in transcriptomic reprogramming. Our study provided a rich resource that facilitates further research on the regulatory mechanisms underlying zebrafish embryonic development and human diseases.

## Results and Discussion

### The Overview of the Developing Transcriptomes

To obtain a genome-wide gene expression profile for early zebrafish development, we constructed nine sequencing libraries for the SOLiD system, with RNAs from major embryonic stages plus an early larval stage (Table 1, Figure S1) [23]. In addition, the ‘60 hpf’ library was sequenced with half depths for a second time (60 hpf-2) to assess technical reproducibility. In total, we obtained over 3 billion reads, out of which ∼58% (∼54% to ∼66% for individual libraries) could be aligned against the zebrafish genome (assembly Zv9; Table 1). Reads with 9 or fewer alignment positions were retained for the estimation of gene expression levels.

**Table 1:** Statistics for reads and detected genes.

Library	Total Reads	Mapped Reads		Genes expressed (RPKM>0.17)
		Uniquely mapped	2–9 locations	
64/128-cell	235,862,240	102,846,478	21,765,138	15,329 (47.4%)
oblong-sphere	338,770,160	162,978,849	28,645,590	14,885 (46.1%)
50%-epiboly	376,278,088	157,933,331	32,136,575	15,508 (48.0%)
15-somite	397,621,313	162,197,274	33,936,889	18,010 (55.7%)
36 hpf	342,026,386	146,707,214	30,287,105	18,774 (58.1%)
48 hpf	378,645,325	156,159,069	34,728,762	18,844 (58.3%)
60 hpf	281,225,289	144,137,292	26,718,211	18,909 (58.2%)
60 hpf-2	99,769,706	49,350,972	9,023,676	18,772 (58.1%)
72 hpf	386,609,124	167,979,863	34,821,945	20,167 (62.4%)
1-week	377,326,926	160,911,108	34,337,779	22,989 (71.1%)

Numbers show sequenced and mapped reads and genes detected as expressed in each library of zebrafish developmental stages. 60 hpf-2, the technical replication for the library of the 60 hpf stage. Percentages in parentheses indicate the proportion of expressed genes in the annotation of the Zv9 genome (Ensembl release 62).

**Figure 1:**
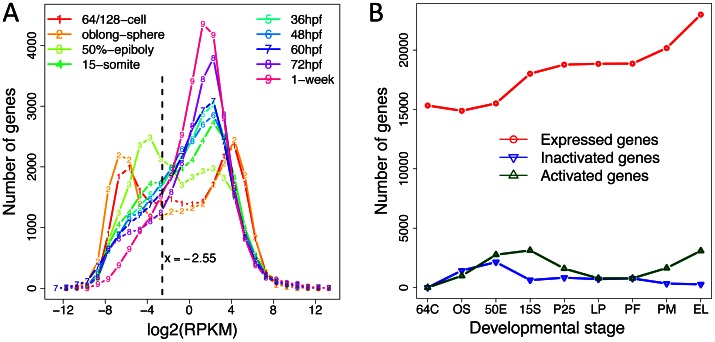
Gene expression profiles across different stages of early zebrafish development. (A) Distribution of RPKM values across the nine developmental stages. The vertical dashed line denotes the threshold above which the genes were determined as expressed. The log2-transformed RPKM values of genes at each stage were binned with interval size 1, and the number of genes falling within each bin was calculated and plotted as a function of log2-RPKM values. (B) Number of genes expressed, activated and inactivated in each developmental stage. Expressed genes at a stage were those with RPKM values above the threshold shown in A; activated genes were defined as those expressed at the present stage but were not expressed at the previous stage, whereas inactivated genes were those expressed at the previous stage but not expressed at the present stage. Stage abbreviations on the x-axis: 64 C, 64/128-cell; OS, oblong-sphere; 50 E, 50%-epiboly; 15 S, 15-somite; 36 h, 36 hpf, prim-25; 48 h, 48 hpf, long-pec; 60 h, 60 hpf, pec-fin; 72 h, 72 hpf, protruding-mouth; 1 w, 1-week, early larva.

Among the 32,312 genes annotated in the Zv9 genome sequence (Ensemble release 62), 27,829 (∼86%) were detected to have at least one read and their expression levels were estimated by calculating the RPKM (reads per kilo base mRNA per millions of mapped reads) values (Table S1). We then examined the distribution of gene expression values for each developmental stage to obtain an overview of the early zebrafish transcriptomes. Both the 64/128-cell and the oblong-sphere stage showed a remarkable bimodal distribution in terms of gene expression, unlike the unimodal distribution for late embryonic and larval stages (Figure 1). Clearly, two main classes of genes existed within embryos at the cleavage, oblong-sphere and possibly 50%-epiboly stages. These two classes might also persist for the following stages, like 15-somite and 36 hpf, yet with lower resolutions. To test whether those lowly expressed genes during the early stages were really expressed, we computed RPKM values for the intergenic regions. We found that, the expression levels of these lowly expressed genes were not higher than that of the intergenic region, thus probably representing background signals (Figure S2). However, these genes were greatly up-regulated during later development, which were in clear contrast to those highly expressed genes, whose expressions were later suppressed (Figure S3). These observations indicated the shift in transcriptome composition during the developmental process.

Our RNA-seq data are of high technical reproducibility, as indicated by the comparison between the two replicated sequencing libraries for the 60 hpf stage. High consistency in the estimation of gene expression levels was observed between the two replicated libraries (r^2^ = 0.9998, Figure S4). We have also performed real-time PCR experiments to validate the expression values for a set of selected genes, and the results were highly consistent with our RNA sequencing data (Figure S5).

### Transcriptome Dynamics and the Underlying Factors during Zebrafish Development

Consistent with the sequential transcriptional activation of the zygotic genome, the number of expressed genes steadily increased during development, with the proportion of expressed annotated genes ranging from 46% to 71% across different libraries (Table 1, Figure 1B). The stages of 50%-epiboly and 15-somite were found to have the largest number of more highly expressed genes comparing to their respective previous stages (Figure 1B). In particular, approximately 2766 genes were elevated in expression (referred as activated hereafter) during the period from mid-blastula transition (MBT; the oblong-sphere stage) to early gastrulation (the 50%-epiboly stage) (Figure S6). The largest number of genes with reduced expression (referred as repressed hereafter) was also observed for the 50%-epiboly stage, suggesting large transcriptomic reconfigurations at this stage.

**Figure 2:**
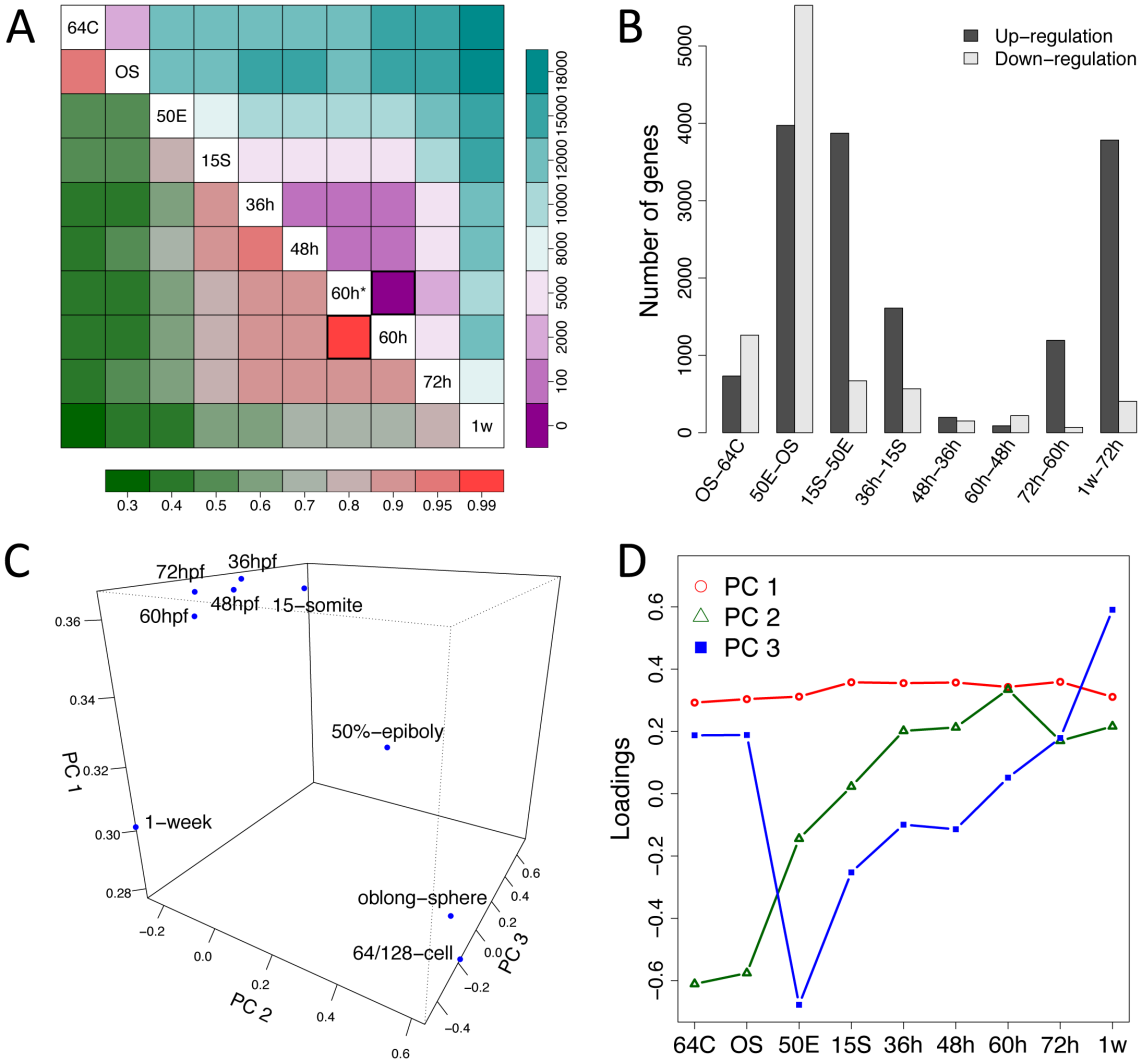
Transcriptome dynamics during early zebrafish development. (A) The correlation of global gene expression profiles and number of differentially expressed genes between developmental stages. Gene expression correlations, as indicated by colors in the bottom triangular, were calculated as the Pearson correlation coefficients using the RPKM values of all the detected genes. Differentially expressed genes were defined as genes with statistically significantly different raw read counts (FDR<0.001) and > = 2-fold changes in the RPKM values between two stages, and their numbers are represented by the colors in the upper triangular. Color bars at the bottom and the right side indicate the scale for correlation coefficients and the numbers of differentially expressed genes, respectively. The two squares with bold borders indicate the correlation and number of differentially expressed genes between the two replicates for the 60 hpf stage. (B) Number of genes significantly up-regulated or down-regulated during developmental stage-transitions. (C) Principal component analysis (PCA) about developmentally regulated genes. The graph illustrates the distribution of each development stage in the space of the first three principal components (PCs). (D) The loading coefficients on the first three PCs for each developmental stage. Stage abbreviations on the x-axes in (B) and (D) are as in Figure 1B.

To more clearly capture the dynamic nature of the transcriptomes during early zebrafish development, we compared the overall gene expression profiles across developmental stages. Expectedly, the similarity in the genome-wide gene expression profiles between different developmental stages generally decreased as the time intervals between them increased (Figure 2A). The Pearson’s coefficient of correlation between any two of the transcriptomes varied from 0.38 to 0.96, with the lowest between those of 64/128-cell and 1-week and the highest between 64/128-cell and oblong-sphere. The coefficients of transcriptomes between adjacent stages were above 0.8 in most cases, but that between the 50%-epiboly and the oblong-sphere was only 0.56, consistent with a large number of regulated genes during this period.

We identified differentially expressed genes between each pair of developmental stages to obtain clues about the developmental regulation of the transcriptomes (see Materials and Methods). In total, we found 16,130 genes (66% of Zv9 genes) showed significantly differential expressions between at least two developmental stages. Not surprisingly, the distribution of the number of differentially expressed genes exhibited a similar pattern to that of the correlation coefficients (Figure 2A). We observed the largest number of differentially expressed genes between the oblong-sphere and the 50%-epiboly among all comparisons of adjacent stages, consistent with the multitude of transcriptional activation/inactivation events during the early gastrula stage (Figure 2B). Meanwhile, a large number of genes were also differentially expressed between the 50%-epiboly and 15-somite stages, indicating that the transcriptomic composition during early gastrulation was rapidly transformed. Functional analysis about these developmentally regulated genes would facilitate the understanding of the molecular events occurring at these important developmental transitions.

We used principal component analysis (PCA) to explore the overall expression pattern among developmental stages. In the space of the first three principal components (PCs), which together explained 93% of the variations in gene expression, the nine stages generally distributed in accordance with their temporal order during embryogenesis (Figure 2C). Notably, the stages of 50%-epiboly and 1-week were separated from their respective preceding and/or subsequent stages, whereas 64/128-cell and oblong-sphere and all the other stages clustered together, respectively. This expression pattern was consistent with the aforementioned results of correlation analysis. Apparently, the differences in gene expression profiles between the embryos of 36 hpf, 48 hpf and 60 hpf were very small, despite the dramatic morphogenetic changes. Examination of the loading of each stage onto the PCs showed that, PC 1 mainly served as the scaling factor, which in fact represents the weighted average of the transcriptional levels across stages (Figure 2D). In contrast, PC 2 and 3 both characterized the differences between stages, with PC 2 representing the differences between the early and later stages and PC 3 representing that between 50%-epiboly and others. Therefore, two major factors might exist to configure the transcriptomes during zebrafish development, with one driving the development process forward and the other representing the considerable reconfiguration at the early gastrulation.

### Clustering of Differentially Expressed Genes Reveals Distinct Expression Patterns for Zebrafish Embryogenesis

**Figure 3:**
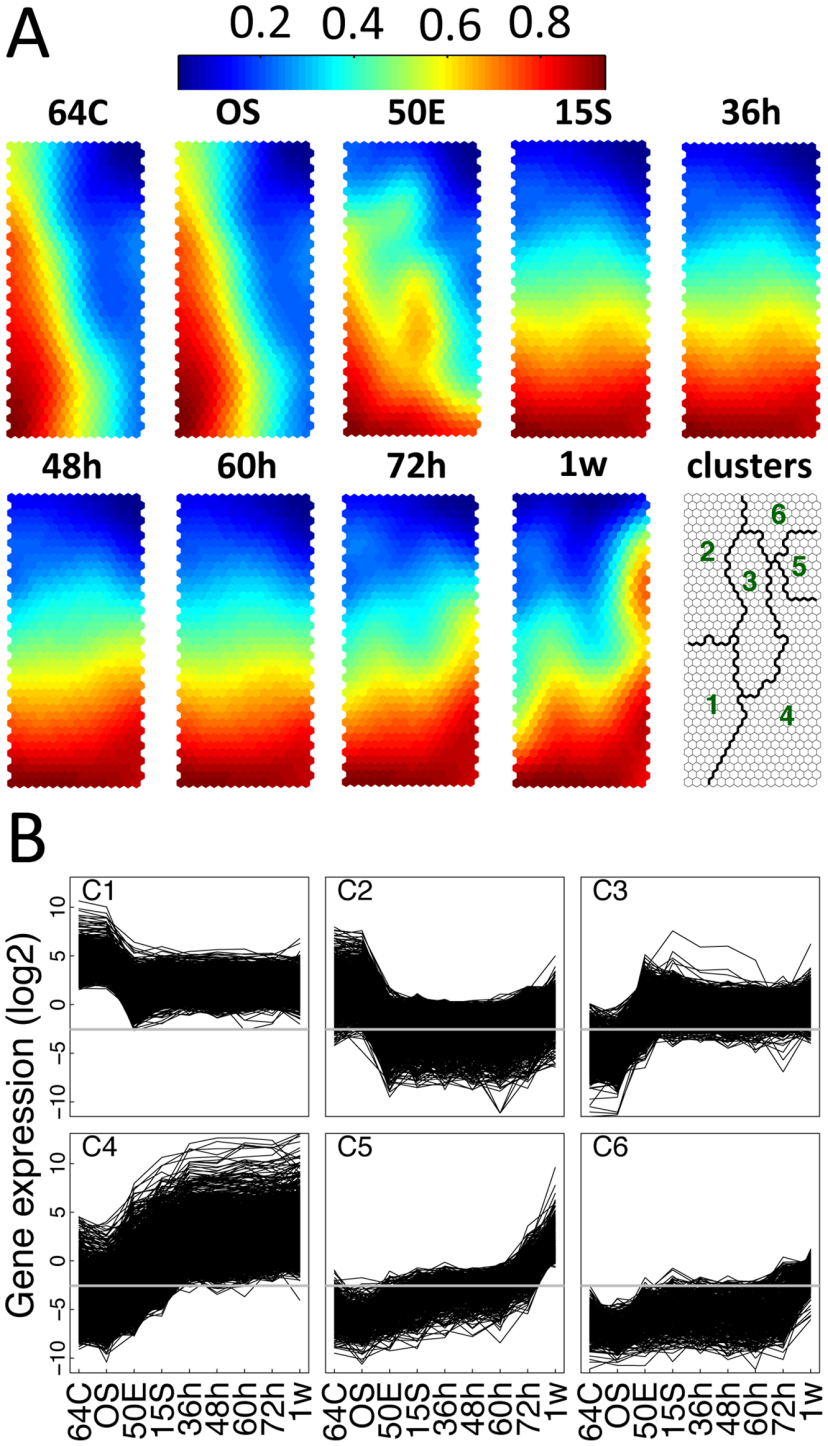
Distinct gene expression patterns in early developing zebrafish. (A) Component planes obtained by self-organization map (SOM) analysis about developmentally regulated genes. Each component plane, except for the one marked as ‘clusters’, illustrates the normalized gene expression for one stage, with colors varying from red via yellow to blue indicating high to intermediate and low expressions. The ‘clusters’ plane shows the clustering of the units on the SOM planes. Map units at corresponding positions in each plane represent the same set of genes, with the number varying from several to hundreds. Clustered regions on the SOM plane represent more coherently expressed genes. Regions are numbered and distinguished by different colors. (B) Line-plots showing gene expression patterns for each identified cluster in A. The expression values are log2-transformed RPKM values. In each sub-graph, marks on the up-left corner denote the respective clusters on the ‘clusters’ plane in A and the grey line demonstrates the expression threshold. Stage abbreviations on the x-axis are as in Figure 1B.

Clustering of genes based on expression patterns is a powerful tool to understand the transcriptome compositions across different samples and/or developmental stages. We employed the self-organizing map (SOM) method for both the clustering and the visualization of the expression patterns represented by different clusters [24], [25]. In this method, genes with highly similar expression patterns are configured into map units that are organized in a manner that the gradual transitions in expression patterns could be visualized on a component plane. Our SOM analysis revealed that the transcriptional patterns during the zebrafish development were transformed in a gradual and stepwise manner, with distinguished patterns observed for most stages (Figure 3A). For instance, the mid-blastula stage showed approximately the same transcriptomic pattern with that of the cleavage stage, whereas both were clearly different from the early gastrula embryos. It is also evident that genes mapped to different regions on the map manifested different transcriptional trajectories throughout the development. In short, genes from the left part of the map were gradually repressed from the cleavage to the gastrula and later stages, whereas those on the right side were activated in different ways during early zebrafish development (Figure 3A).

To more clearly explore the expression patterns revealed by the SOM analysis, we performed a clustering analysis on the SOM outputs to merge map units with similar expression patterns into larger clusters. We obtained six such clusters, each of which contained hundreds to thousands of genes sharing a distinct expression pattern (Figure 3, Table S2). The first two clusters (C1 and C2) mainly contain genes repressed from the mid-blastula stage onwards, with the two distinguished to each other by the average expression levels across developmental stages. Most genes of C1 were almost constantly expressed above a threshold level from the early gastrula stage onwards, while most of the C2 genes were expressed below this threshold level. This indicated that, despite the sharp down-regulation after MBT, most genes in C1 probably still function during the later stages, which might not be true for most C2 genes. GO enrichment analysis showed that, genes functioning in mitosis and methylation processes were overrepresented in C1, whereas those involved in meiosis and piRNA metabolic processes were enriched in C2 (Table S2 and Table S3). Further functional studies could help us better understand the biological implications of these two different inactivation modes for cleavage and/or mid-blastula preferential genes. Genes in C3 showed peak expressions at the early gastrula or 15-somite stages, although they may also be significantly up-regulated during the later stages of embryogenesis. Genes with such expression patterns include those participating in heart looping, hematopoietic stem cell proliferation, leukocyte migrations and left/right pattern formation (Table S3).

The last three clusters contain genes up-regulated during the embryogenesis, albeit with different tempos and modes. C4 genes are activated very early, dramatically and rapidly up-regulated from the mid-blastula till the 15-somite and/or 36 hpf stages and persisted high expression throughout the subsequent stages. Genes of this cluster were enriched for the processes of DNA dependent transcription, Notch signaling pathway as well as canonical Wnt receptor signaling pathway (Table S3). A large amount of genes participating in developmental processes of multicellular organisms was also found in this cluster, such as anterior/posterior and dorsal/ventral pattern formation, nervous system development, gonad development, embryonic hematopoiesis, otic placode formation as well as cartilage development (Table S3). Unlike C4, C5 mainly consists of genes activated very late, whose expressions were only dramatically up-regulated at or after 72 hpf. C5 could also be called 1-week larva preferential because over 80% genes (1011/1257) in this cluster showed 1-week larva-preferential expression (see below). The most notable functional gene groups in C5 were those involved in visual perception, phototransduction, DNA mediated transposition and xenobiotic metabolic process (Table S3). Genes in C6 were expressed far below the threshold expression level during most time of the embryogenesis, only starting to be up-regulated at around the early larva stage. Enriched biological pathways among C6 genes included homophilic cell adhesion, immune response and rhythmic process (Table S3). This cluster also contained genes whose expression could only be detected in the 1-week larva fish, including those participating in the telencephalon and diencephalon cell migration, organ regeneration, maintenance of DNA methylation, immune response and visual learning. Therefore, these processes and developmental events are likely not initiated until the beginning of larval growth.

### Genes Preferentially Expressed at Each Developmental Stage

**Figure 4:**
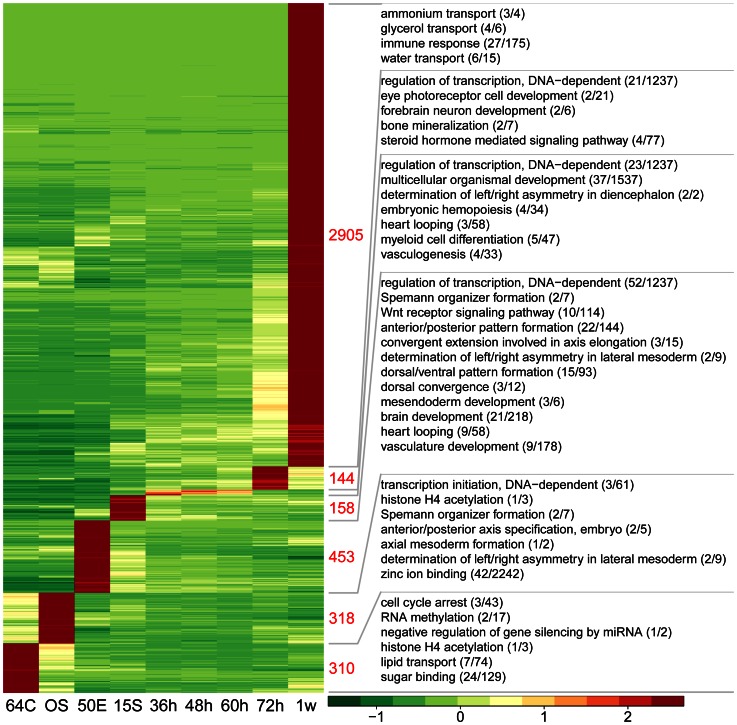
Genes preferentially expressed in one developmental stage of zebrafish. The heatmap at the left side visualizes the self-division of preferentially expressed genes of each stage. A gene was taken as preferentially expressed at one stage when it has both significantly higher numbers of detecting reads (FDR<0.001) and higher RPKM values (> = 2-fold) at this stage compared with that at any other stages. In the graph, color gradient illustrates the z-scores of the expression values of the genes, which were calculated as the mean-centered RPKM values divided by the standard deviation for each gene, separately. Right to the heatmap shown the number of preferential genes and selected significantly enriched GO terms (Fisher’s exact test *P*<0.05, see texts for details) for the corresponding stages indicated by the grey lines. Numbers in parentheses represent genes annotated to the corresponding GO terms in the preferentially expressed gene group and the genome, respectively. Stage abbreviations below the heatmap are as in Figure 1B.

Genes with significantly higher expression at one developmental stage than in any other stages were considered to be preferentially expressed at that stage, called stage-preferential genes hereafter (see Methods). These genes could serve as molecular signatures to distinguish the developmental stages from each other, since they are most likely involved in stage specific developmental processes. We identified 4294 stage-preferential genes in total, with the 1-week larva fish expressing the largest number of these stage-preferential genes (2905), followed by the embryos at the early gastrulation, mid-blastula and cleavage stages (Figure 4, Table S4). We found few or none stage-preferential genes for the embryos at 36 hpf, 48 hpf and 60 hpf. This result suggested the transitional nature of the embryonic transcriptomes during these periods, where most of the expressed genes were in the middle of transcriptional activation or inactivation. To find if there were any collective signature genes for these three stages, we searched for and found only 30 genes whose minimal expressions at these stages were significantly higher than that of any other stages (Table S4).

We next performed the Gene Ontology (GO) enrichment analysis to explore the overrepresented biological pathways and molecular functions among each set of the stage-preferential genes. The results revealed numerous developmental or molecular events well known to occur at the respective embryonic stages, as well as many previously unknown ones (Figure 4 and Table S5 and S6). The preferential genes of the 64/128-cell stage were enriched for biological processes important for cleavage, such as histone acetylation, lipid transport, cell cycle arrest, and G2/M transition DNA damage checkpoint, in accordance with previous findings [26], [27]. Unexpectedly, genes involved in sugar binding (GO:0005529; n = 24) were also enriched among 64/128-cell preferential genes, suggesting unknown yet critical sugar-related pathways during cleavage. Similarly, it is also unexpected that genes involved in zinc ion binding (GO:0008270; n = 46) were over-represented among the oblong stage preferential genes, cluing us about the critical nature of maintaining zinc status during MBT. The analysis also provided insights into the roles of many genes including TGF-beta activated kinase 1/MAP3K7 binding protein 3, CREB binding protein a, MYC-associated zinc finger protein during MBT. Other biological processes enriched among MBT preferential genes included DNA-dependent transcription initiation, Spemann organizer formation, determination of left/right asymmetry in lateral mesoderm, positive regulation of fibroblast growth factor receptor signaling pathway, anterior/posterior axis specification and embryo axial mesoderm formation, well concordant to previous reports [28]. These good agreements between our GO enrichment results and existing experimental findings suggested the reliability of our data, whereas the unexpected enrichment of processes such as sugar binding and ion binding at particular stages clued us for unknown mechanisms during embryogenesis.

Biological processes enriched among 50%-epiboly preferential genes were found to be very important for gastrulation, such as dorsal/ventral pattern formation, anterior/posterior pattern formation, convergent extension involved in axis elongation, mesendoderm development, dorsal convergence, brain development, Wnt receptor signaling pathway and vascular development, which is again in accordance with previous studies [28] (Figure 4 and Table S5). For instance, the genes *sizzled*, *chordin*, *bmp2b* and *bmp7a* were identified as 50%-epiboly preferential genes, conforming to their demonstrated functions in the specification of dorsal-ventral polarity by modulating BMP2 and BMP7 activity [28]–[30]. Likewise, another 50%-epiboly preferential gene, *nodal-related2*, has implicated functions in the establishment of dorsal-ventral polarity during gastrulation and later on the patterning of nervous system [31]. Meanwhile, the early gastrula preferential expression of several genes, including *tll1*, *polm*, *bambia* and *fzd8,* provided evidence for their involvement in axial patterning. In addition to body plan patterning, critical steps of brain development, such as forebrain/hindbrain boundary formation and neurogenesis, also occur during early gastrulation. Among the group of genes annotated to brain development (GO:0007420, n = 21), several encode factors with verified brain development functions, like Foxa2, Irx7, Flh, Has2, Sp5l and Frzb [28], [32]–[36], whereas more await further investigation.

Among the genes preferentially expressed at the 1-week stage (n = 2905), those involved in visual perception (GO:0007601; n = 24) and immune response (GO:0006955; n = 27) were significantly enriched (Figure 4 and Table S5). Several of the visual perception related gene have already been characterized. For instance, knockdown of the gene encoding the cone-specific receptor kinase GRK7a in larval zebrafish leads to impaired cone response recovery and delayed dark adaptation [37], and deficiency of *arr3a* causes a severe delay in photoresponse recovery which, under bright light conditions, is rate-limiting [38]. Similarly, it has been demonstrated that loss of function of the cone-specific gene *pde6c* results in rapid cone photoreceptor degeneration in zebrafish, providing a good model for developing therapies of related diseases [39]. We noted that several genes with postulated participation in visual perception, like *gprc5c*, *prph2a*, *prph2b*, *opn4.1* and *opn4xb*, were also preferentially expressed at 1-week. The functions of these genes in visual perception architecture development remain to be understood.

Zebrafish is also a good model system for studying mechanisms of adaptive immune response since many components of the immune system are conserved between zebrafish and human [40]. Highlighting this advantage, several conserved immune responsive factors were found as 1-week-stage preferentially expressed. For instance, β2-microglobulin is required for the full expression of xenobiotic-induced systemic autoimmunity in human [41], which might be conversed in zebrafish since it has been shown in the large yellow croaker that β2-microglobulin could be required for the antiviral immune response triggered by poly I:C [42]. Similarly, the zebrafish gene *interleukin-15* may also participate in immune response, since the exon-intron organization of this gene is conserved among zebrafish, mammals and chicken and the human counterpart has been implicated in the development of NK cells [43], [44]. Notably, some of the genes annotated for immune response have no characterized functions, including *si:dkey-200l5.2*, *si:ch211-149o7.4*, *zgc:153067*, *si:dkeyp-2h4.2* and *zgc:101788*. Further investigations are required to determine their roles in immune response.

### Distinct Expression Patterns among and within Transcription Factor Families

Transcription factors (TFs) are important regulators of a variety of cellular processes. Identification of the transcription factors active during embryogenesis is an important step in understanding the underlying gene regulatory networks. Currently, the zebrafish genome (Zv9) has 1916 genes annotated to encode transcription factors (TFs), which could be classified into 68 different families plus two groups with unknown family identities [45]. We found 1567 TF genes that were expressed in at least one of the stages examined here (Table S7). Leaving the five genes with unknown family identity, the remaining 1562 genes belong to 67 families, uncovering expression for members from nearly all families.

**Figure 5:**
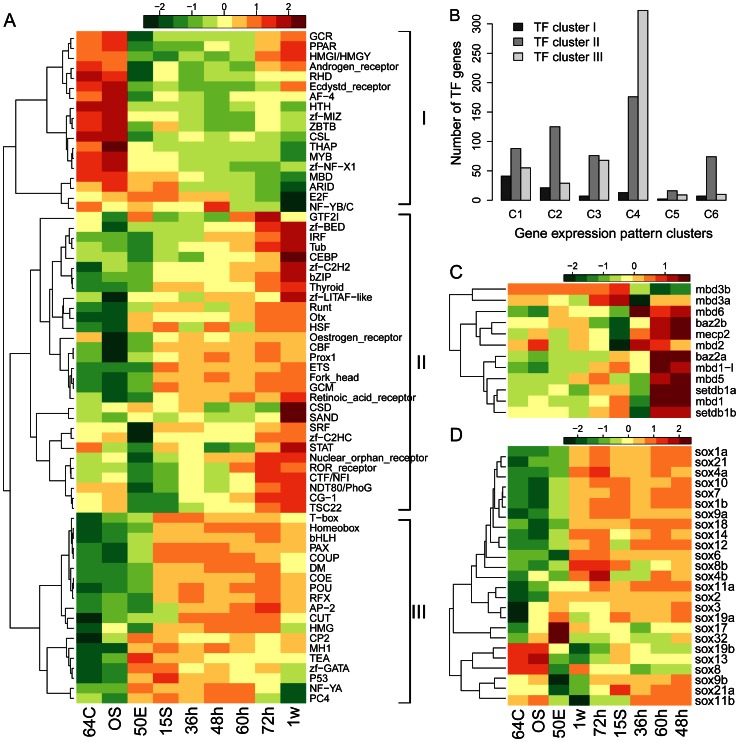
The distinct expression patterns of transcription factors in early developing zebrafish. (A) The expression patterns of transcription factor (TF) families expressed during early development of zebrafish. Shown are the log2-transformed median expression values for each TF family. The TF families are clustered into three groups, each with distinct expression patterns, as marked by the Roman numbers. (B) Comparison of the clusters of TF families and the clusters of developmentally regulated genes. Labels at the x-axis denote clusters identified by the SOM analysis in Figure 3. (C) The expression patterns of the MBD gene family. (D) The expression patterns of the Sox genes. Stage abbreviations on the x-axes are as in Figure 1B.

We performed clustering analysis about these TF families, to identify whether any of them share similar expression patterns. We found that they could be classified into three clusters with distinctive expression patterns (Figure 5A). Cluster I has 18 families, whose average expression levels decreased significantly after MBT, although enhanced expression could also be observed in 72 hpf embryos and 1-week larva in several cases. Cluster II and III shared similar expression profiles, containing 30 and 19 TF families respectively. Both clusters of families have enhanced average expression levels from the 50%-epiboly or 15-somite stages onwards, concordant with the genome-scale transcription initiation at these stages. However, the nadir and zenith of the expression of cluster II families seemed to be one phase later than that of cluster III families (Figure 5A). In particular, most families in cluster III had their minimal transcript abundance observed in the cleavage and mid-blastula embryos, compared with the minimal abundance for most of the cluster II families occurring in the mid-blastula or early gastrula stages. Similarly, most cluster II families reached their peak expressions at the 72 hpf and 1-week stages, whereas most cluster III families achieved their maximal expressions at the 72 hpf or earlier stages. These interesting expression patterns for different TF families await further exploration to better understand the gene functional network underlying embryonic development.

Despite the characteristic expression patterns, members from each TF family also showed substantial diversity in transcriptional dynamics. The three clusters of TF families comprise 184, 1120, 605 genes respectively, among which 170, 802 and 590 were expressed at one or more developmental stages and 91, 555 and 494 were significantly differentially expressed between developmental stages. It seems genes from the third TF cluster are under more delicate regulations during embryogenesis. Moreover, most of the differentially expressed TF genes showed expression patterns characteristic of their respective TF clusters, as demonstrated by comparison with the gene expression patterns revealed by our SOM analysis (Figure 5B). It is also evident that a significant portion of genes in each TF cluster showed atypical expression dynamics in regard to the characteristic patterns.

The expression patterns of and within each TF family are closely related to their functions. In particular, the dramatic down-regulation of the cluster I families at the 50%-epiboly stage might be required for the transcriptional activation of the zygotic genome. This was well demonstrated by the expression patterns of genes encoding the Methyl-CpG binding domain (MBD) containing proteins. The MBD domain has the ability to specifically bind methyl-CpG nucleotides, although in some cases this ability has been lost [46], [47]. By binding to methylated DNA, MBD proteins play pivotal roles in translating the DNA methylation status to chromatin structure remodeling and final gene transcription regulations. Twelve MBD genes have been annotated in the Zv9 genome, all of which were detected by our sequencing (Figure 5C). Two closely related paralogs, *mbd3a* and *mbd3b*, showed peak expression at the early gastrula stage. These two genes might play important roles in establishing proper gene expression patterns during gastrulation and/or organogenesis, probably through their involvement in the nuclear remodeling and histone deacetylation complex [48]. In contrast, the expression of three other genes was markedly decreased at early gastrulation but then restored to moderate expressions during later embryonic development. One of these genes, *mecp2*, is the founder of the MBD family and extensively studied. The inactivation of *mecp2* during early gastrulation is consistent with its postulated roles of transcriptional repression at specific developmental stages and with the large-scale transcription activation of the zygotic genome after MBT [46]. The reactivation of *mecp2* during somitogenesis and pharyngula stages suggested it might be required for normal neurological development and mature neurons in adult animals [49]. All the other *MBD* genes generally decreased in expression during development, including *mbd1* and *setdb1b*, which had similar expression patterns and could form a complex to establish the gene repressive marker of H3K9 methylation [50], [51].

The large cohort of TF families constituting the clusters II and III might play active roles during embryogenesis and organogenesis, such as the homeobox (Hox) gene family and the high mobility group box (HMG-box) family. Hox genes are central regulators in the development of body patterning in vertebrates, conserved across diverse species [52]. About 300 functional Hox genes have been identified in the zebrafish genome [53], and we were able to obtain the expression information for 273 of them (Figure S7). Forty-three Hox genes were found to be stage-preferential genes, suggesting involvement in stage-specific functions (Table S8). Seventeen of the 43 stage-preferential genes show maximal expression at the 50%-epiboly stage, including *cdx4, cdx1a* and *otx1a* (Figure S8). *cdx4* and *cdx1a* act redundantly to regulate other Hox genes which is crucial for patterning all the three embryonic germ layers [54]. Our observation about their consistent peak expression during early gastrulation corroborated this point. The observed preferential expression of *otx1a* in 50%-epiboly embryos was concordant with its demonstrated roles in vertebrate brain regionalization during gastrulation [55], [56]. Like *otx1a*, all the other four Otx proteins, constituting a paired-like homeobox gene family, were also expressed in the embryos and larvae of zebrafish (Figure S8B). For example, *crx* and *otx5* shared very similar expression patterns and both peaked at 72 hpf, consistent with their regulatory relationships and the roles in retinal specification and differentiation [57], [58]. Several of the Three Amino acid Loop Extension (TALE)-class Hox genes were preferentially expressed in 1-week larvae, including *pknox1.1*, *pknox1* (Ensembl Gene ID: *ENSDARG00000018765*) and *pbx4b* (Ensembl Gene ID: *ENSDARG00000091547*) (Figure S8A). The TALE-class Hox proteins, including *pbx4*, *pbx2* and *meis1* can form complexes and/or function as cofactors of other Hox proteins, such as *hoxa1*, *hoxb7a*, *hoxa9a*, regulating various processes like hematopoiesis and hindbrain development [59]. Unlike the almost constant high expression of *pbx4*, *pbx4b* persisted low expression and dramatically up-regulated at the 1-week stage. Surprisingly, *pbx4b* and *pbx4* encode proteins with identical sequences, suggesting their divergence mainly occurs at the regulatory level. On the other hand, the two close tandem paralogous genes *pknox1.1* and *pknox1* shared very similar expression patterns and might function redundantly with each other (Figure S8A). Interestingly, two Hox genes, *prop1* and *gsx1*, showed significantly higher expression in the three stages 36 hpf, 48 hpf and 60 hpf than in any other stages (Figure S8A). *prop1* is a conserved regulator of pituitary gland development in vertebrates [60] and *gsx1* is considered to specify the boundaries of hypothalamus and intermediate spinal cord during somitogenesis [61]. Their high expression at the pharyngula, hatching and pec-fin periods suggested they might have unknown functions that were crucial for organ development during the later stages of embryogenesis.

The HMG-box containing proteins have been implicated in the regulation of numerous DNA-dependent processes such as transcription, replication, DNA repair and site-specific recombination [62]. Proper expressions of HMG-box genes are required for the progression of embryogenesis whereas mutations in the HMG-box domains cause severe developmental defects and cancers [63]–[65]. We detected the expression for all but one of the 57 known HMG-box genes during zebrafish development. Most of the HMG-box genes showed an enhanced expression during early gastrulation, which was maintained throughout the subsequent stages of embryogenesis (Figure S9). Nearly half of the HMG-box family encode the SRY related (Sox) proteins, which are engaged in diverse embryonic developmental processes, including sex determination, heart and brain development as well as homeostasis in adult tissues [66]–[68]. The zebrafish genome contains at least 26 Sox genes, all but one of which was likely expressed during embryogenesis (Figure 5D). Two Sox genes, *sox13* and *sox19b*, were highly expressed at the 64/128-cell and oblong-sphere stages but decreased during later development, indicating their critical roles for the cleavage and mid-blastula embryos. Dramatic up-regulation during the several earliest stages was observed for fifteen Sox genes, whose high abundance persisted throughout the later stages. Two other genes, *sox9b* and *sox11b*, already reached high expression at the 64/128-cell stage. These genes might play essential roles for MBT, gastrulation and organogenesis. Interestingly, *sox17* and *sox32* showed complement expression patterns with *sox9b* and *sox21a*, with the former two genes preferentially expressed during early gastrulation and the latter two extremely depleted in the early gastrulation compared to all the other stages. How these genes relate to the gastrulation process awaits further investigations.

### Expression Dynamics of the Wnt Signaling Pathway

Our transcriptome data could provide a solid foundation to advance our understanding about signaling pathways playing crucial roles during embryonic development. Among the numerous pathways regulating development, the Wnt signaling pathway is of particular importance because of its indispensability in the coordination of various cell behaviors such as cell movement, cell proliferation and stem cell maintenance, the involvement in developmental processes such as the body axis formation and organogenesis, and the conservation of Wnt signaling components across the animal kingdom [69], [70]. Deregulation of critical Wnt molecules might result in severe developmental defects and diseases; therefore, Wnt molecules have been taken as important therapeutic and pharmacology targets [71]–[73].

**Figure 6:**
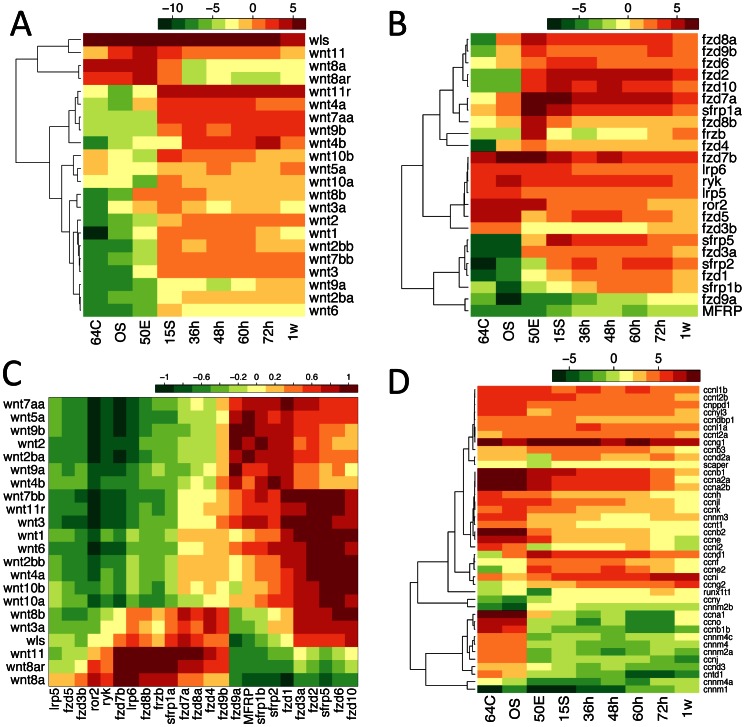
The expression patterns of the Wnt signaling pathway and the cyclin genes. (A) and (B) show the log2-transformed expression values of the Wnt gene family and the Wnt receptor genes, respectively. (C) The similarity and dissimilarity in the expression patterns between the Wnt genes and Wnt receptors. Color gradient represents Pearson’ correlation coefficients of the expression values. (D) The log2-transformed expression values of the genes encoding cyclines. Stage abbreviations on the x-axes are as in Figure 1B.

The Wnt signaling process is initiated by the binding of Wnt proteins to the cognate cell surface receptors. The Zv9 genome contains at least 26 Wnt genes, among which 22 were detected by our RNA sequencing (Figure 6A). One of these genes, *wls* (*wntless homolog*), was highly and constantly expressed throughout embryogenesis, consistent with previous reports and its role in the regulation of Wnt protein secretion [74]. Among the other Wnt genes, nineteen were found to be differentially expressed between developmental stages, indicating that they might be subject to stage-specific regulations and therefore functioned in stage-dependent manners.

Three Wnt genes showed high or increasing expression till early gastrulation. Among these genes, *wnt11* is well known for its involvement in the non-canonical Wnt signaling where a downstream β-catenin independent pathway was stimulated upon the binding of Wnt11 to its receptors [75], [76]. Due to its key roles in mediating convergent extension cell movement, Wnt11 has been implicated in various developmental processes including the body axis formation, cardiogenesis and neuronal differentiation [76]–[78]. In amphibians, Wnt11 forms a complex with Wnt5a to determine the dorsal-ventral axis formation [79]. However, in zebrafish, it is Wnt8a that specifies the dorsal-ventral axis [80]. The data shown here confirm the maternal identity for *wnt8a*. Furthermore, a close paralog of *wnt8a*, called *wnt8a-related* (*wnt8ar*) here, was highly similarly expressed as *wnt8a*, suggesting they may have partially redundant roles although functional divergence might also exist.

All the other genes showed enhanced expression during or after early gastrulation (Figure 6A), most notably *wnt11r*, which is highly similar to *wnt11* in sequence. After early gastrulation, *wnt11* was dramatically reduced, whereas *wnt11r* was constitutively highly expressed, supporting the proposition that *wnt11r* in fish and amphibians is the functional homolog of the mammalian *wnt11* [81]. In addition, *wnt4* was similarly highly expressed as *wnt11r*, in agreement with the redundant roles between Wnt11, Wnt11r and Wnt4 in the control of the midline assembly of organ precursors [82].

Currently, over 20 Wnt receptors have been identified in zebrafish, most of which belong to the Frizzled (Frz) family [83]. We therefore examined the expression dynamics of these receptors and performed a comparison with that of Wnt genes (Figure 6B). Some receptors likely had maternal origins, including two Frz genes, *Fzd5* and *Fzd7b*, and all the four non-Frz receptors, since they were highly expressed till the early gastrulation. Most of the other Frz genes started to increase in expression during or after MBT. The expression patterns shown here were consistent with previous findings, as in the cases of *fzd7a* [84], *Fzd7b* [85] and *sfrp1* [86].

Expression pattern comparison between Wnt genes and receptors showed clear-cut division of Wnt-Receptor pairs into two classes, with high positive correlations observed mainly for within-class pairs (Figure 6C). This was a direct reflection of the fact that most Wnt genes and receptors were either maternally supplied (reduced after early gastrulation) or zygotically transcribed (increased in expression during embryogenesis). Although high expression correlations do not always imply co-localization or physical interactions, we indeed observed that the ligand-receptor cognation or co-localization between Wnt11 and Fzd7a/b, Ryk, Ror2 proteins coincide with their highly similar expression patterns [83], [87]. In cases the expression correlations did not match reported ligand-receptor cognations, a possible explanation could be that the interactions require other co-factors which function in a stage- and tissue-specific manner. Our results thus supported the proposition that Wnt factors and receptors perform their functions depending on specific cellular contexts [70].

### Implications for Novel Functions of Known genes Involved in Embryonic Development

Our high-throughput sequencing data also provided clues about previously unknown embryonic roles for genes with already known functions. One example is the *klf4* gene, which has been shown to be expressed from 70% epiboly onwards and play crucial roles in primitive erythropoiesis and anterior mesendoderm/pre-polster differentiation [88], [89]. Our data indicated that *klf4* was activated much earlier than previously thought. During MBT, the transcript abundance of *klf4* was strikingly elevated to a level 80-fold higher than that at the cleavage stage, and reduced slightly during early gastrulation and somitogenesis, finally to a significantly lower level during later development. This suggested *klf4* might carry out unknown functions and/or that the biological processes involved by *klf4* were in fact initiated earlier than previously recognized.

We have also examined the expression dynamics of cyclin genes during embryogenesis. Cyclins are a family of proteins controlling cell cycle via interactions with cyclin-dependent kinases [90]. We detected the expression of 41 out of the 45 cyclin genes annotated in the Zv9 genome (Figure 6D). During development, most of these genes were gradually down-regulated, especially after MBT, consistent with the reduction of the cell division rate after the transition from the synchronous cell cycles with only S and M phases to the asynchronous cell cycles that also contain G1 and G2 phases [23]. This class of genes included the ones with well-documented maternal origins, such as *ccnb1* and *ccne* [91], [92]. In addition to genes with gradual reduction in expression, several other cyclin genes showed increased expression, persistent high abundance or stage-specific up-regulation during embryogenesis. One of such genes is *cnnd1*, whose protein product, cyclin D1, has been shown to be responsible for G1/S transition and whose knockdown impaired eye and head development [93]. Here we found that *cnnd1* was markedly up-regulated during early gastrulation and slowly decreased thereafter, congruent with its characterized roles during embryogenesis. The most highly and almost constantly expressed cyclin gene, *ccng1*, has been shown in mammals to be transactivated by the tumor suppressor gene product p53 and to induce cell cycle arrest or apoptosis upon DNA damage [94]–[96]. The transcriptional regulation by p53 and the involvement in cell cycle control of *ccng1* seem to be conserved in zebrafish, although the detailed functions during embryogenesis remain unclear [97], [98]. Another gene, *ccni*, previously identified from human brain [99], was continuously elevated in expression level during development. Notably, several genes were specifically down-regulated during early gastrulation, including *runx1t1* and *scaper*. *scaper* encodes a cyclin A homolog and has been demonstrated to be involved in modulating the functions of cyclin A/Cdk2 in S and M phase transitions in mammals [100]. Hence, the depletion of *scaper* transcripts during early gastrulation in zebrafish was very likely associated with the shift from short S/M cell cycles to the full cell cycles.

### Conclusions

Transcriptome analysis is a powerful tool to study genome-wide gene activities, providing rich resources for understanding the mechanisms underlying numerous biological phenomena. Zebrafish is a popular model animal for the study of biological processes as important as vertebrate development and adaptive autoimmune responses. In this study, we employed RNA sequencing to further approach the transcriptome dynamics during early zebrafish development. The vast amount of gene expression data provided us a comprehensive view about the rapidly reprogrammed transcriptomes of early developing zebrafish. A large number of genes were found to be differentially expressed between developmental stages, and could be classified into six clusters with distinct expression patterns. Lots of stage preferentially expressed genes were also identified, whose functions might be very important for normal progress of vertebrate development. Analysis about transcription factors, signaling pathways and other important gene families suggested that, our study could help to advance our understanding about the mechanisms of vertebrate development as well as the causes of numerous human diseases.

## Materials and Methods

### Ethics Statement

Zebrafish used in this study were maintained in the zebrafish facility of Shanghai Research Center for Model Organisms (SRCMO), in accordance with IACUC-approved protocol (SRCMO-IACUC No: 2009-0001).

### Collection of Zebrafish Embryos and Larvae

Zebrafish embryos and larvae were collected and maintained at approximately 28.5°C, and were staged according to their morphological features and hours (h) or days (d) post-fertilization as described previously [23]. Nine stages covering seven different developmental periods were used: (1) cleavage (64/128-cell), (2) blastula (oblong-sphere), (3) gastrula (50%-epiboly), (4) segmentation (15-somite), (5) pharyngula (36 hpf), (6) hatching (48 hpf, 60 hpf, 72 hpf) and (7) early larval stage (1-week). As rapid cooling is a method of euthanasia in zebrafish [101], liquid nitrogen rapid cooling methods were used for zebrafish embryos and larvae euthanasia in this work.

### RNA Isolation

Total RNA was extracted from the aforementioned stages of embryos or larvae using Trizol (Invitrogen, Carlsbad, CA, USA) methods according to the manufacturer’s instruction. RNA quality was evaluated by gel electrophoresis, with the concentration measured with NanoDrop 2000 (Thermo Scientific, Waltham, MA, USA). The aliquots were stored at −80°C. Five hundred nanograms of total RNA were used in each amplification reaction.

### mRNA-seq library Preparation and Sequencing

We didn’t separate the embryonic samples into different replicates before mRNA-seq library construction, considering sequencing costs. However, by using a large number of embryos (∼2000 per stage), we were essentially assaying the average expression profile for each gene across a variety of different biological individuals, allowing us to obtain a good representative transcriptome for zebrafish embryos and larvae.

We performed mRNA-seq according to a protocol/kit available from Life Technologies, with minor modifications described as follows. Briefly, the total RNA was estimated on an Agilent 2100 Bioanalyzer (Agilent Technologies, Waldbronn, Germany). The ribosomal RNA-removal steps were replaced by two rounds of polyA purification. None of the samples showed signs of degradation or impurities (260/280 and 260/230>1.8, RIN >8.5).

The total mRNA was first purified using the PolyATtract® mRNA Isolation Systems (Promega, Madison, WI, USA), followed by another round of purification using the Poly(A)Purist™ Kit (Ambion, Austin, TX, USA). The removal of ribosomal RNAs was confirmed on an Agilent 2100 Bioanalyzer. About 0.8 µg of mRNA was fragmented with 10 min at 37 ^o^C RNase III treatment. The fragmented mRNA were ligated with adaptor Mix A, which were subsequently used for reverse transcription. The first strand cDNA were separated using 6% TBE-Urea Gel (Invitrogen, Carlsbad, CA, USA) and 100–200 nt fraction was recovered. The fractionated cDNA were subjected to 11–15 cycles of PCR amplification, with the PCR products purified to yield the SOLiD Fragment Library ready for emulsion PCR. Emulsion PCR was performed using 600 pg of the library. All experiments were performed on full sequencing slides. Fifty-base sequences were obtained using SOLiD sequencing (ABI, Foster, CA, USA).

### Real-time RT-PCR

To verify the mRNA-seq data, twelve genes with different expression patterns were tested using real time RT-PCR for the nine developmental stages. Reverse transcription was carried out with M-MLV Reverse Transcriptase (Promega, Cat.No.M1705, Madison, WI, USA) using oligo d(T) primers. Real-time PCR was performed with an EvaGreen dye (Biotium, Cat. No. 31000, San Francisco Bay Area, CA, USA) on MJ DNA Engine OpticonTM System (PTC-200 DNA EngineTM Cycler and CFD-3200 OpticonTM Detector, NewYork, NY, USA). The expression of β-actin1 was used as an endogenous control and amplified with Primer-F (CATCCGTAAGGACCTGTATGCCAAC) and Primer-R (AGGTTGGTCG TTCGTTTGAATCTC). Primers for the twelve verified genes are listed in Table S9.

### Mapping of Reads and Calculation of Gene Expression Level

The short reads obtained by the SOLiD sequencing were aligned against the zebrafish genome assembly version 9 (Zv9; http://www.sanger.ac.uk) using the Bioscope software package supplied by the Life Technologies company. Bioscope uses a seed-and-extend approach to map reads against the reference. At the seeding phase, one or multiple anchor seeds subtracted from the read will be mapped against the reference, to determine whether the alignment will proceed at all. Specifically, for 50 bp reads, the seeds are 25 bp long and at most two mismatches to the reference are allowed. Extension begins from the anchor position of the seed, to both directions, if possible. During extension, a base match with the reference will receive a score of 1 and a mismatch gets a penalty of −2, by default. By this scheme, the ultimate alignment is the one with the highest score across the genome and when the alignments are tied in terms of the score, the shortest one will be selected. The algorithm also maps the reads against all known or putative splice junctions. A splice junction alignment will be accepted if any end of the spliced exons overlap with the read by at least 8 bp. The penalty score for alignment to putative splice junctions is −1. A read is taken as uniquely aligned if its best alignment score is larger by 5 than that of the secondary alignment score. Aligned reads were then assigned to genes using an in-house developed Perl script (available upon request), with the gene models downloaded from the Ensembl project website (release 62 for Zv9; http://www.ensembl.org/info/data/ftp/index.html). Briefly, a read was assigned to a gene if it was aligned against one of the exons of any annotated transcript variants of the gene, or if it fallen within one of the potentially expressed introns. Potentially expressed introns of a gene were defined as introns with normalized read counts that were above 80% of the normalized read counts of the joined annotated exons of that gene, where the count for an intron was normalized against its length and the counts for exons were aggregated and normalized against the total length of all annotated non-overlapping exonic regions. Reads overlapped with introns by more than 10 nucleotides were taken as intron-originated.

Reads for a gene was summarized as follows: each unique read was counted as 1, whereas reads with multiple alignment locations were equally split between all the locations, adding the inverse of the number of locations to the count of the respective gene. The normalized gene expression level was calculated as reads per kilo-base of mRNA length per millions of mapped reads (RPKM) (PMID: 18516045) for each library separately. The transcript length for a gene was calculated as the length of exonic regions plus the length of the potentially expressed intronic regions. To determine the expression threshold of a gene at a stage, we calculated RPKM values for each intergenic region separately, with reads positioned within 500 bp to any end of any gene excluded. By assuming the normalized read counts of the genes in the late developmental stages follow a distinct distribution from that of the intergenic regions, we set the expression threshold as 0.17 RPKM, which is the maximal likelihood estimation of the threshold classifying genomic regions into two parts, i.e. expressed and unexpressed (Figure S2).

### Statistical Analysis

We identified differential expression for each gene between any two libraries using the Fisher’s exact test, by taking the raw number of reads of the genes as input. False discovery rates (FDRs) were then calculated using the p-values of the Fisher’s exact tests, as proposed in [102]. We determined one gene as significantly differentially expressed between two libraries when the FDR was less than 0.001 and the absolute fold change in RPKM values was larger than 4. A gene was defined as preferentially expressed in one stage, if for this gene all the FDRs of the comparisons performed between that stage and any other stages were <0.001 and the RPKM value at that stage was at least two times larger than at all the other stages.

GO annotation data were downloaded from Ensembl using the BioMart tool. In total, we obtained 99,666 GO term-gene linkages for 21,969 genes. We adopted the R package topGO to evaluate the significance of enrichment of each GO term within an interested set of genes, using the default algorithm and Fisher’s exact test [103]. The advantage of this package is that, it can decorrelate the hierarchical structure of the GO term system and effectively remove the local dependencies between GO terms, which in most cases enabled us to find out the most specific terms relevant to the addressed biological questions.

We used the SOM method for both clustering and visualization of the expression patterns of genes differentially expressed between developmental stages [24], [25]. Using the SOM Toolbox developed in the Matlab computing environment, we fitted SOM to histogram-equalized log2-transformated RPKM values for all genes across all developmental stages. By histogram-equalization, the log2-transofrmed RPKM values were ordered and almost equally divided into a determined number of bins, with the original values linearly transformed in each bin. A final linearization scaled the transformed values to [0,1]. The original RPKM values could be recovered by the inverse of the above process. Hierarchical clustering of the units in the SOM was subsequently performed using the hclust function implemented in the R software [104], followed by manual adjustment, to ensure that, within-cluster units shared coherent and characteristic expression patterns. In each cluster of units, the top half of the genes more tightly associated with the center of the expression profiles were used for line-plots.

Heatmap and the associated clustering were performed using custom-tailored heatmap.2 function from the R package gplots [105].

## Supporting Information

Figure S1 **Images for the zebrafish embryos and larvae of the selected stages.** 64/128-cell stage, belonging to the cleavage period, ∼1.5 hpf; Oblong-sphere stage, belonging to blastula period, ∼3.6–4 hpf; 50%-epiboly stage, belonging to the gastrula stage, ∼5.25 hpf; 15-somite stage, belonging to segmentation period, ∼17 hpf; 36 hpf or prim-25 stage, belonging to pharyngula period; 48 hpf or long-pec stage, belonging to hatching period; 60 hpf or pec-fin stage, belonging to hatching stage; 72 hpf or protruding-mouth, belonging to hatching stage; 1-week larva.
(TIF)

Figure S2 **Comparison of RPKM values of genes in the early and late developmental stages and that of the intergenic regions.** The blue line indicates the distribution of RPKM values of the intergenic region during the development, whereas the green and red lines represent distribution of RPKM values for genes at the first two stages and the last five stages analyzed, respectively. The intergenic regions were defined as genomic regions that are far from any end of any by >500 bp. The vertical dashed line indicates the log2-transformed expression threshold (RPKM_0_) we have determined.
(TIFF)

Figure S3 **Expression profiles of genes with background and high expression levels in the early zebrafish embryos.** Heatmap showing the detailed expression patterns (A, B) or z-scores of RPKM values (C, D) for genes with high expressions (A, C) or background expression signals at the 64/128-cell and oblong-sphere stages, respectively. In each graph, the vertical boxes indicate the distribution of the z-scores of the RPKM values for genes at each developmental stage. For each box, the upper and lower borders indicate the first and third quartile points, whereas the bold line shows the median of the z-scores of the respective stage; the whisker at each side extend to the distance 1.5 times the inter-quartile range from each border and the circles outside the whiskers indicate outliers.
(TIF)

Figure S4 **Comparison of gene expression values for the two sequencing replications of the 60** **hpf stage.** X-axis and y-axis represent log2-transformed RPKM values obtained by sequencing runs on the full and half slide of the SOLiD 3.0 platform, respectively. Red and blue points indicate genes with significantly different expression measures between the two runs, while grey points denote genes with statistically the same measures. The r^2^ value shows the coefficient of determination between the two replications of RPKM values.
(TIF)

Figure S5 **Validation of RNA-seq data by real-time RT-PCR.** For each gene, the original RPKM values, the relative RPKM values and the real-time qRT-PCR derived relative expression levels are shown. For each gene, the relative RPKM values were computed as the ratios of the normalized RPKM values to the maximal normalized RPKM value of that gene, where the normalized RPKM values were the ratios of the original RPKM values to that of the *β-actin 1* gene. The y-axes at the left side indicate the RPKM values, whereas those at the right side indicate the relative RPKM and qRT-PCR derived expression levels. The upward triangles and the red line indicate the RPKM values, the open circles and the blue line represent the relative RPKM values, while the solid circles and the green line show the relative expression levels obtained by qRT-PCR. The good consistency between relative RPKM values and the qRT-PCR derived expression levels suggests that our RNA-seq data are well validated.
(TIF)

Figure S6 **Number of genes activated/inactivated per minute at developmental stage transitions.** The upward and downward triangles represent the number of activated and inactivated genes per minute at each stage transition, respectively. For each stage, activated genes are genes expressed at that stage but not at the previous stage, whereas inactivated genes are genes expressed at the previous stage but not at the current stage. The time interval for each stage transition were calculated as the difference in the onset time of the corresponding stages determined by Kimmel *et al.*, 1995.
(TIF)

Figure S7 **The expression patterns of the homeobox genes** **during the early development of zebrafish.** Colors indicate the log2-transformed RPKM values for each gene at each developmental stage. The dendrogram was obtained using the clustering algorithm ‘complete linkage’ based on the distances of the cosine metric.
(TIFF)

Figure S8 **The detailed expression of selected homeobox genes** **during the early development of zebrafish.** In each graph, x-axis indicates the developmental stages while the y-axis shows the RPKM values for each gene on the log2 scale. Color and symbols are used to differentiate genes. (A) Eight homeobox genes that are discussed in the manuscript. Notably, both *pbx4b* and *pknox1* have very few expressions and functional data and have not been formally named before this study. (B) The Otx-class homeobox genes.
(TIFF)

Figure S9 **The expression of the HMG-box genes during the early development of the zebrafish.** The color gradient shows the log2-transformed RPKM values for HMG-box genes at each developmental stage, as indicated by the colorbar. The dendrogram was obtained using the clustering algorithm ‘complete linkage’ based on the distances of the cosine metric.
(TIFF)

Table S1 Gene expression information for zebrafish development.
(XLS)

Table S2 Custering of developmentally regulated zebrafish genes.
(XLS)

Table S3 GO terms of the biological process (BP) category that are significantly enriched among each cluster of developmentally regulated zebrafish genes.
(XLS)

Table S4 Genes preferentially expressed at each developmental stage.
(XLS)

Table S5 GO terms of the BP catergory that are enriched among genes preferentially expressed in one developmental stage.
(XLS)

Table S6 GO terms of the molecular function (MF) catergory that are enriched among genes preferentially expressed in one developmental stage.
(XLS)

Table S7 Transcription factors expressed during early development of zebrafish.
(XLS)

Table S8 Homeobox genes that are preferentially expressed in one developmental stage of zebrafish.
(XLS)

Table S9 Primer sequences of all validated genes.
(XLS)
