# Supplementary figures and images for: Deep mRNA Sequencing Analysis to Capture the Transcriptome Landscape of Zebrafish Embryos and Larvae
Source: PLoS One. 2013 May 20;8(5):e64058. doi: 10.1371/journal.pone.0064058 (PMC3659048; doi:10.1371/journal.pone.0064058)

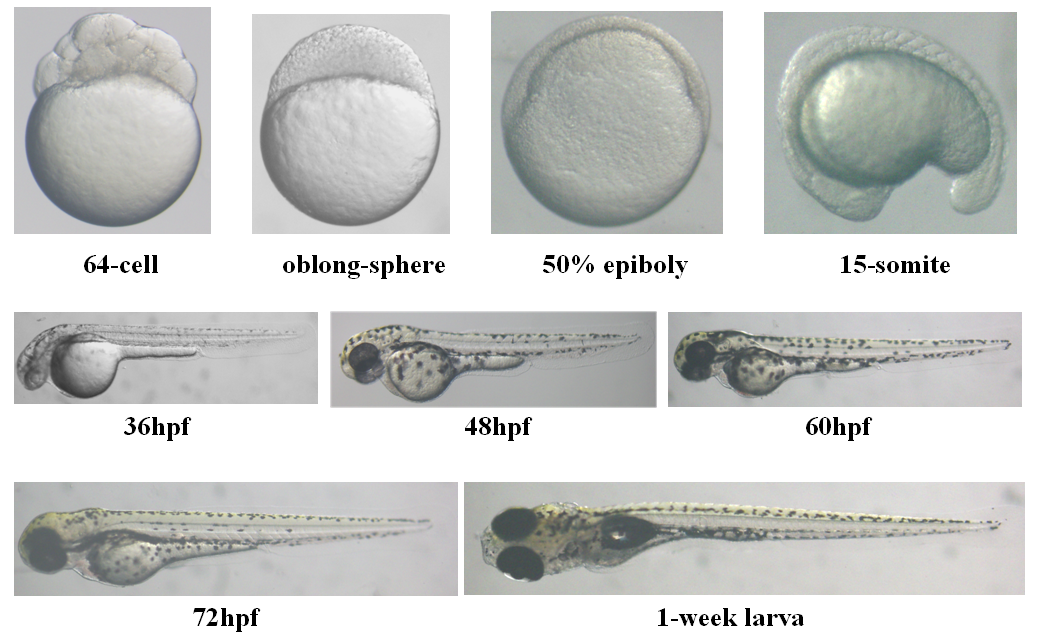

Supplement: Figure S1 — Images for the zebrafish embryos and larvae of the selected stages. 64/128-cell stage, belonging to the cleavage period, ∼1.5 hpf; Oblong-sphere stage, belonging to blastula period, ∼3.6–4 hpf; 50%-epiboly stage, belonging to the gastrula stage, ∼5.25 hpf; 15-somite stage, belonging to segmentation period, ∼17 hpf; 36 hpf or prim-25 stage, belonging to pharyngula period; 48 hpf or long-pec stage, belonging to hatching period; 60 hpf or pec-fin stage, belonging to hatching stage; 72 hpf or protruding-mouth, belonging to hatching stage; 1-week larva. (TIF) [file pone.0064058.s001.tif]

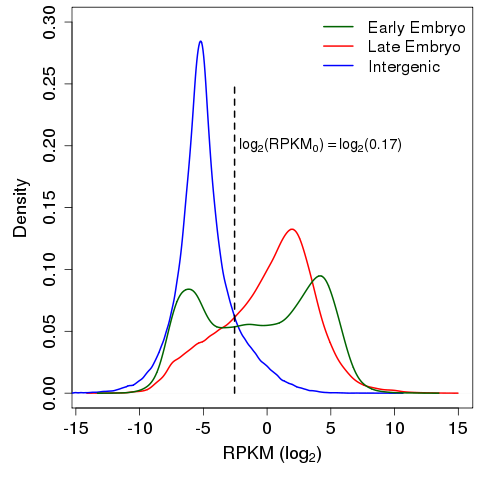

Supplement: Figure S2 — Comparison of RPKM values of genes in the early and late developmental stages and that of the intergenic regions. The blue line indicates the distribution of RPKM values of the intergenic region during the development, whereas the green and red lines represent distribution of RPKM values for genes at the first two stages and the last five stages analyzed, respectively. The intergenic regions were defined as genomic regions that are far from any end of any by >500 bp. The vertical dashed line indicates the log2-transformed expression threshold (RPKM0) we have determined. (TIFF) [file pone.0064058.s002.tiff]

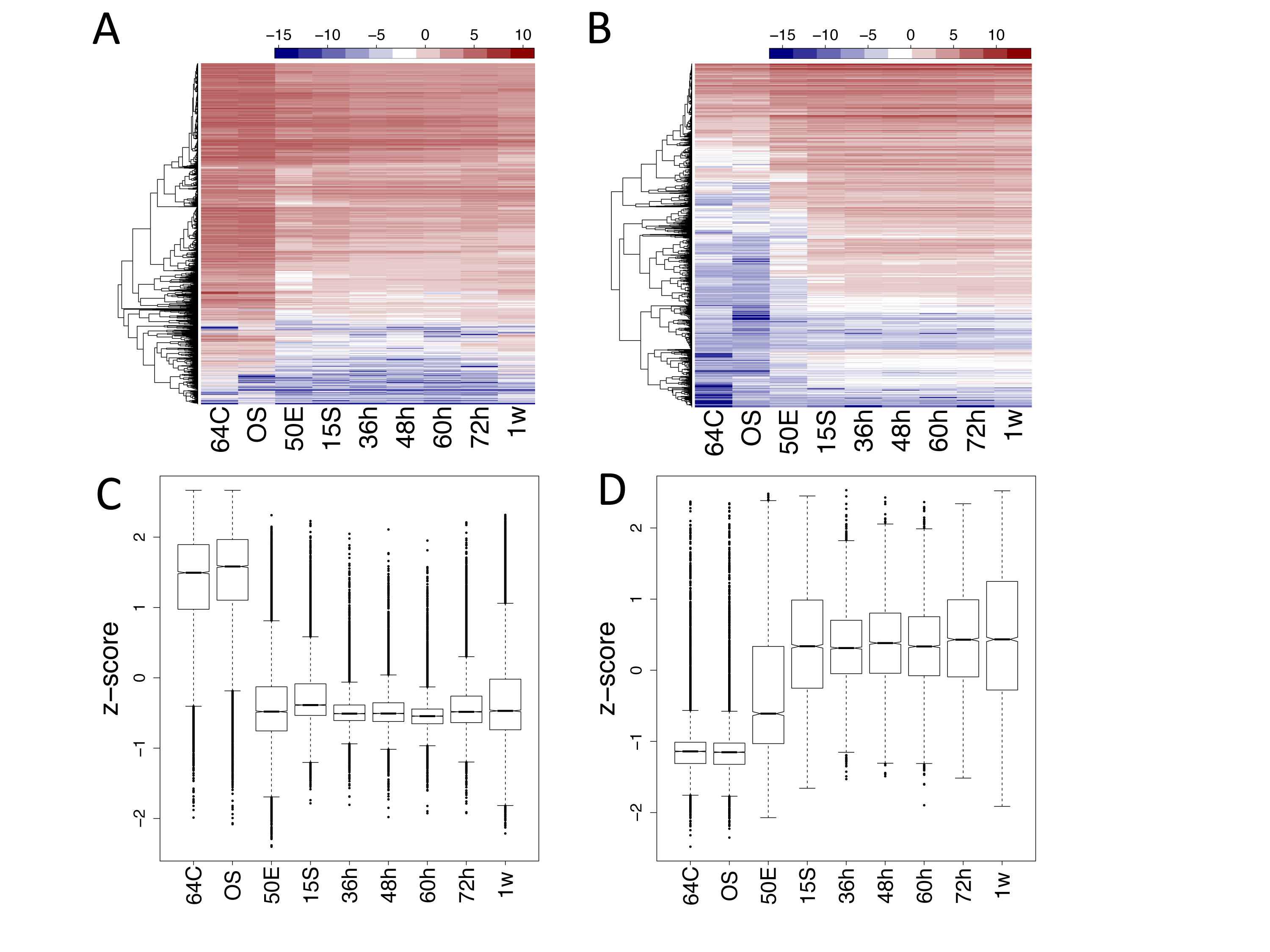

Supplement: Figure S3 — Expression profiles of genes with background and high expression levels in the early zebrafish embryos. Heatmap showing the detailed expression patterns (A, B) or z-scores of RPKM values (C, D) for genes with high expressions (A, C) or background expression signals at the 64/128-cell and oblong-sphere stages, respectively. In each graph, the vertical boxes indicate the distribution of the z-scores of the RPKM values for genes at each developmental stage. For each box, the upper and lower borders indicate the first and third quartile points, whereas the bold line shows the median of the z-scores of the respective stage; the whisker at each side extend to the distance 1.5 times the inter-quartile range from each border and the circles outside the whiskers indicate outliers. (TIF) [file pone.0064058.s003.tif]

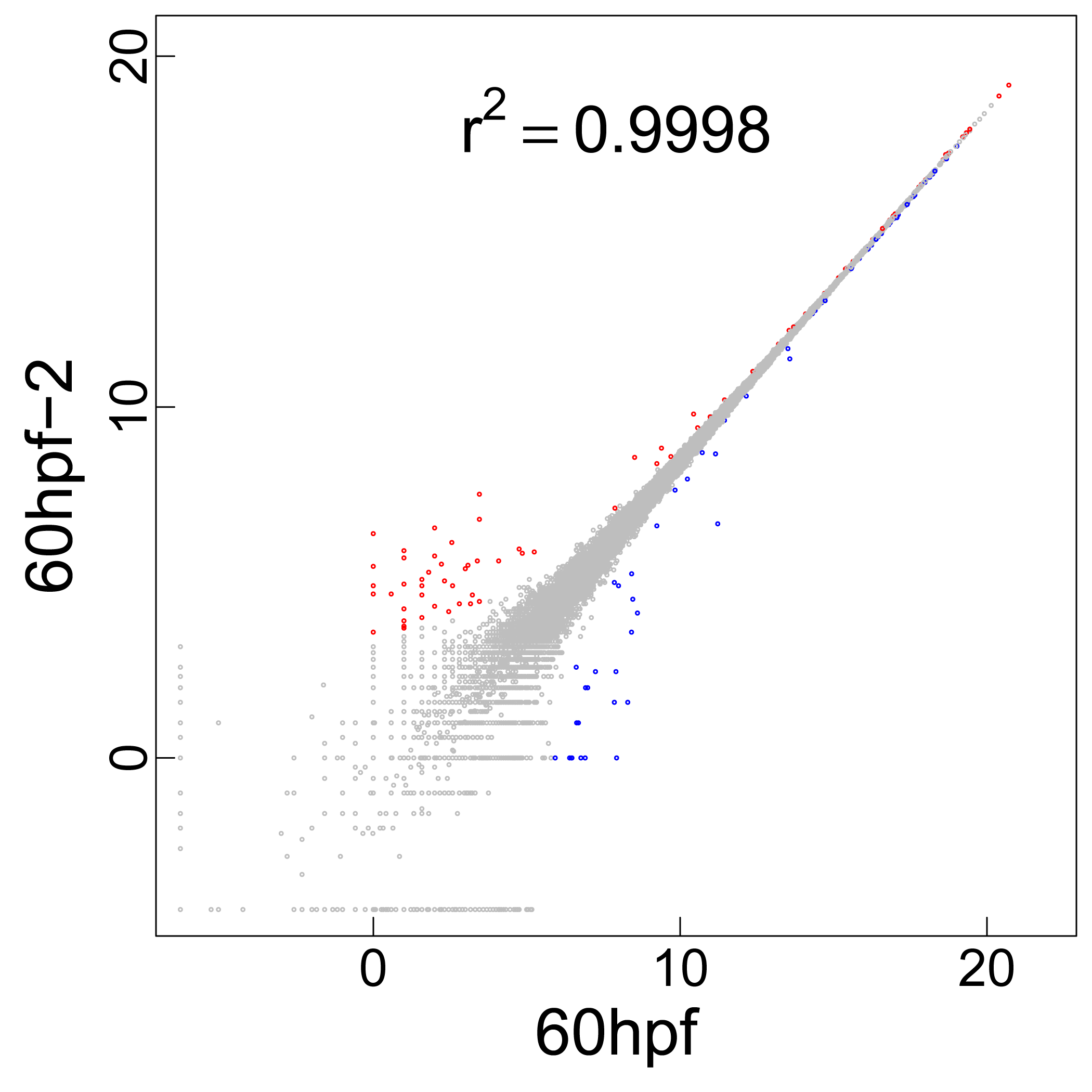

Supplement: Figure S4 — Comparison of gene expression values for the two sequencing replications of the 60 hpf stage. X-axis and y-axis represent log2-transformed RPKM values obtained by sequencing runs on the full and half slide of the SOLiD 3.0 platform, respectively. Red and blue points indicate genes with significantly different expression measures between the two runs, while grey points denote genes with statistically the same measures. The r2 value shows the coefficient of determination between the two replications of RPKM values. (TIF) [file pone.0064058.s004.tif]

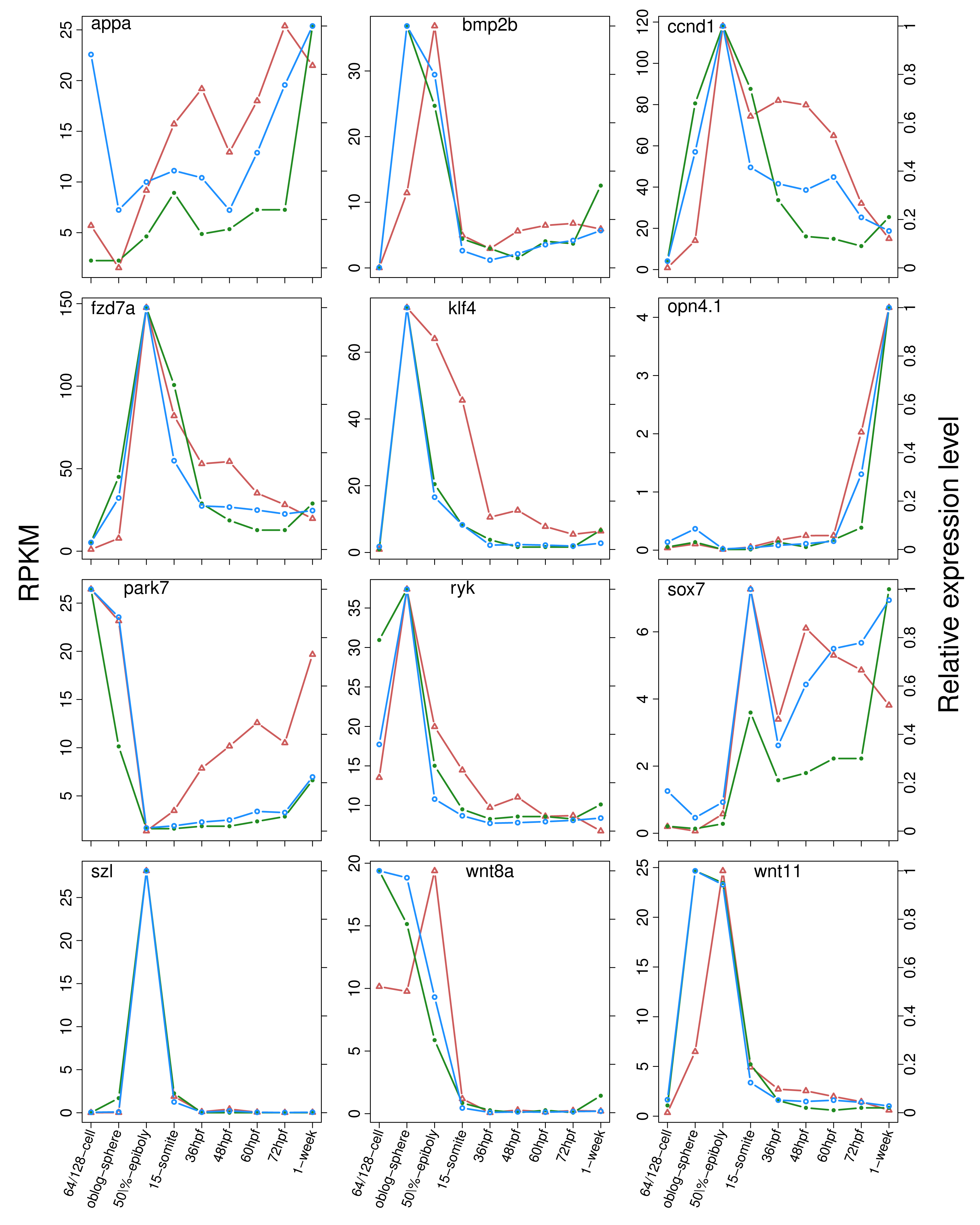

Supplement: Figure S5 — Validation of RNA-seq data by real-time RT-PCR. For each gene, the original RPKM values, the relative RPKM values and the real-time qRT-PCR derived relative expression levels are shown. For each gene, the relative RPKM values were computed as the ratios of the normalized RPKM values to the maximal normalized RPKM value of that gene, where the normalized RPKM values were the ratios of the original RPKM values to that of the β-actin 1 gene. The y-axes at the left side indicate the RPKM values, whereas those at the right side indicate the relative RPKM and qRT-PCR derived expression levels. The upward triangles and the red line indicate the RPKM values, the open circles and the blue line represent the relative RPKM values, while the solid circles and the green line show the relative expression levels obtained by qRT-PCR. The good consistency between relative RPKM values and the qRT-PCR derived expression levels suggests that our RNA-seq data are well validated. (TIF) [file pone.0064058.s005.tif]

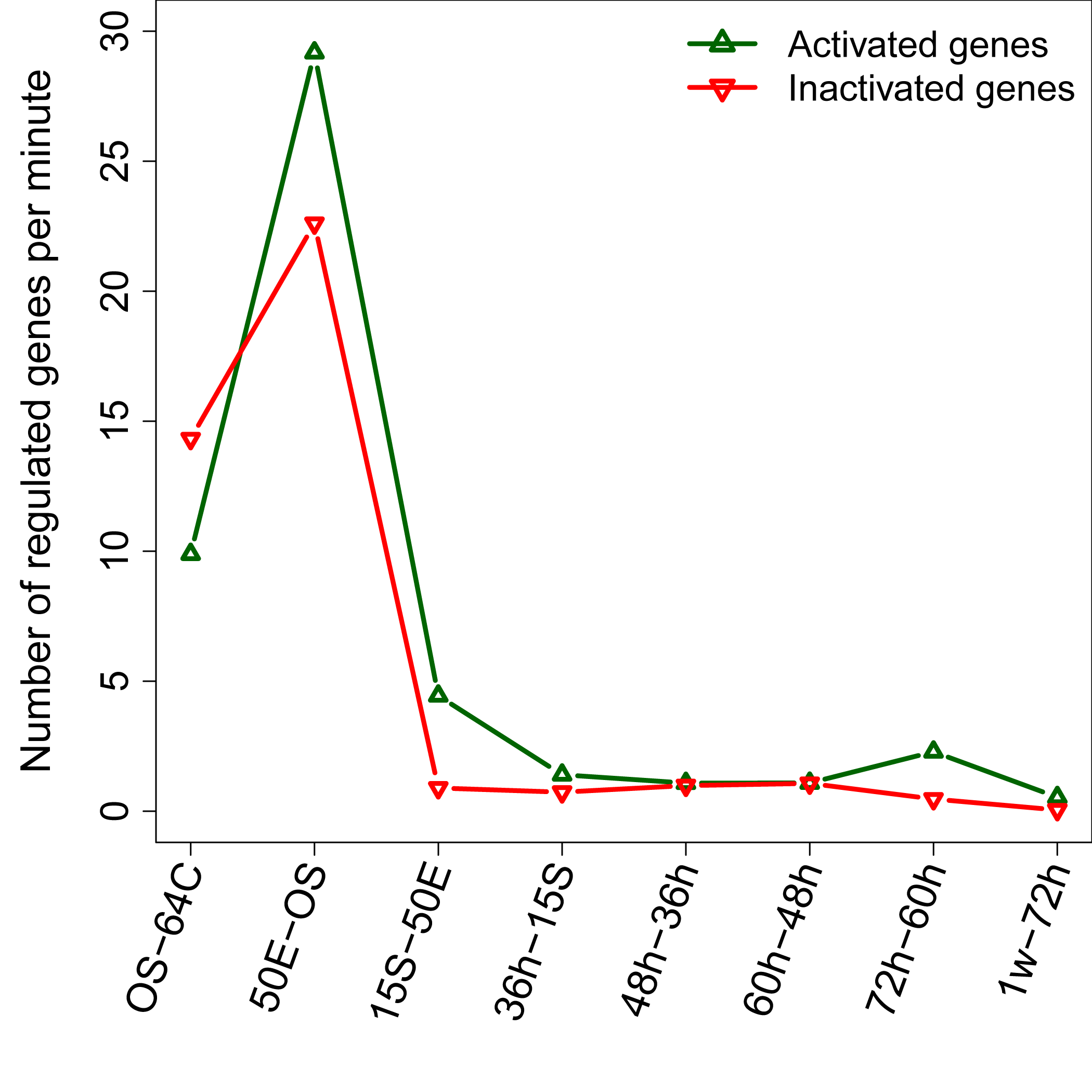

Supplement: Figure S6 — Number of genes activated/inactivated per minute at developmental stage transitions. The upward and downward triangles represent the number of activated and inactivated genes per minute at each stage transition, respectively. For each stage, activated genes are genes expressed at that stage but not at the previous stage, whereas inactivated genes are genes expressed at the previous stage but not at the current stage. The time interval for each stage transition were calculated as the difference in the onset time of the corresponding stages determined by Kimmel et al., 1995. (TIF) [file pone.0064058.s006.tif]

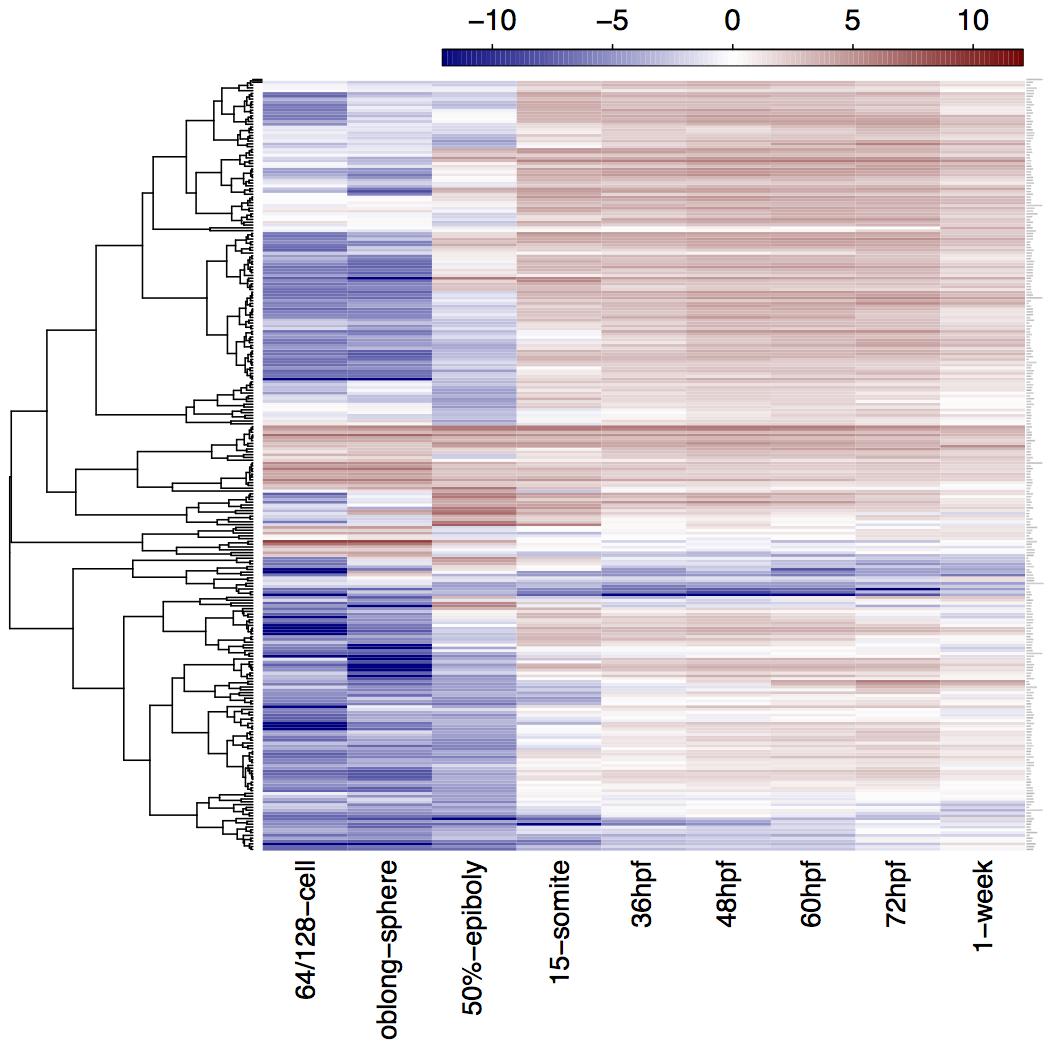

Supplement: Figure S7 — The expression patterns of the homeobox genes during the early development of zebrafish. Colors indicate the log2-transformed RPKM values for each gene at each developmental stage. The dendrogram was obtained using the clustering algorithm ‘complete linkage’ based on the distances of the cosine metric. (TIFF) [file pone.0064058.s007.tiff]

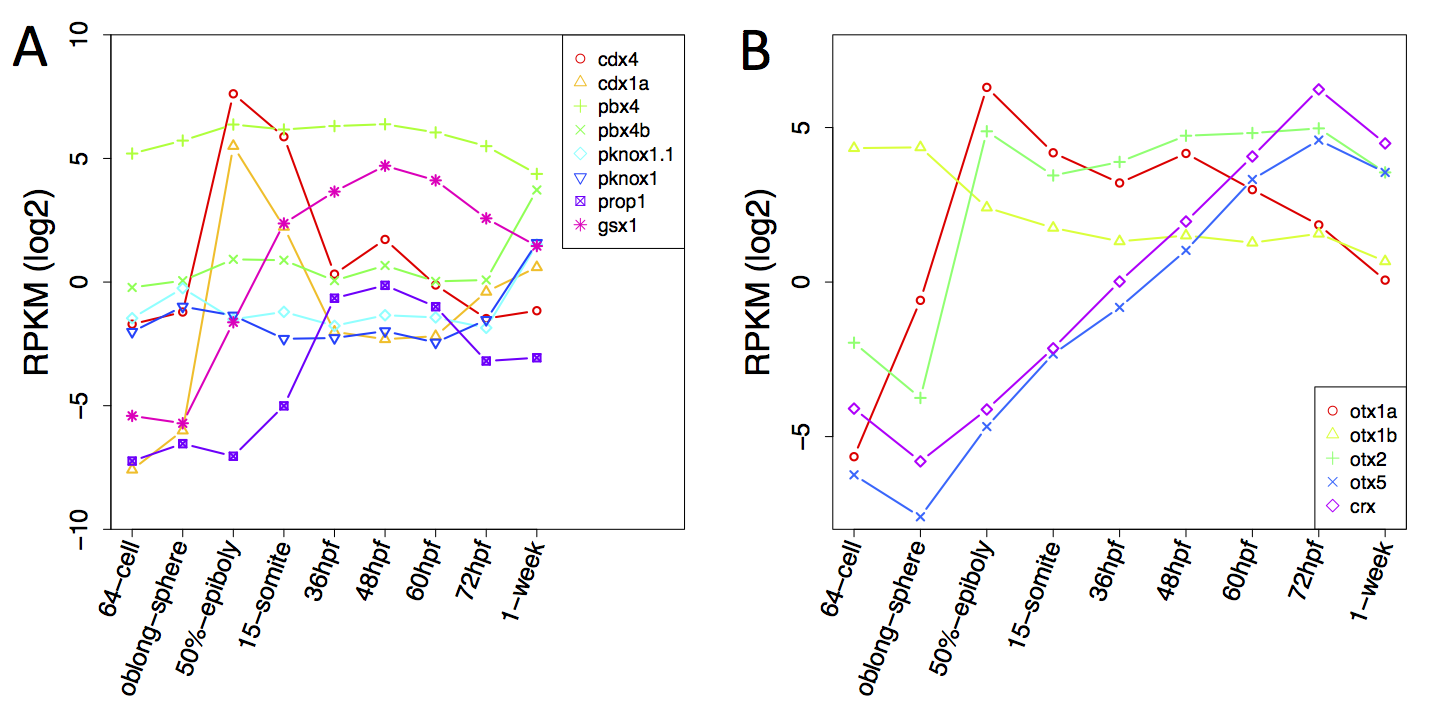

Supplement: Figure S8 — The detailed expression of selected homeobox genes during the early development of zebrafish. In each graph, x-axis indicates the developmental stages while the y-axis shows the RPKM values for each gene on the log2 scale. Color and symbols are used to differentiate genes. (A) Eight homeobox genes that are discussed in the manuscript. Notably, both pbx4b and pknox1 have very few expressions and functional data and have not been formally named before this study. (B) The Otx-class homeobox genes. (TIFF) [file pone.0064058.s008.tiff]

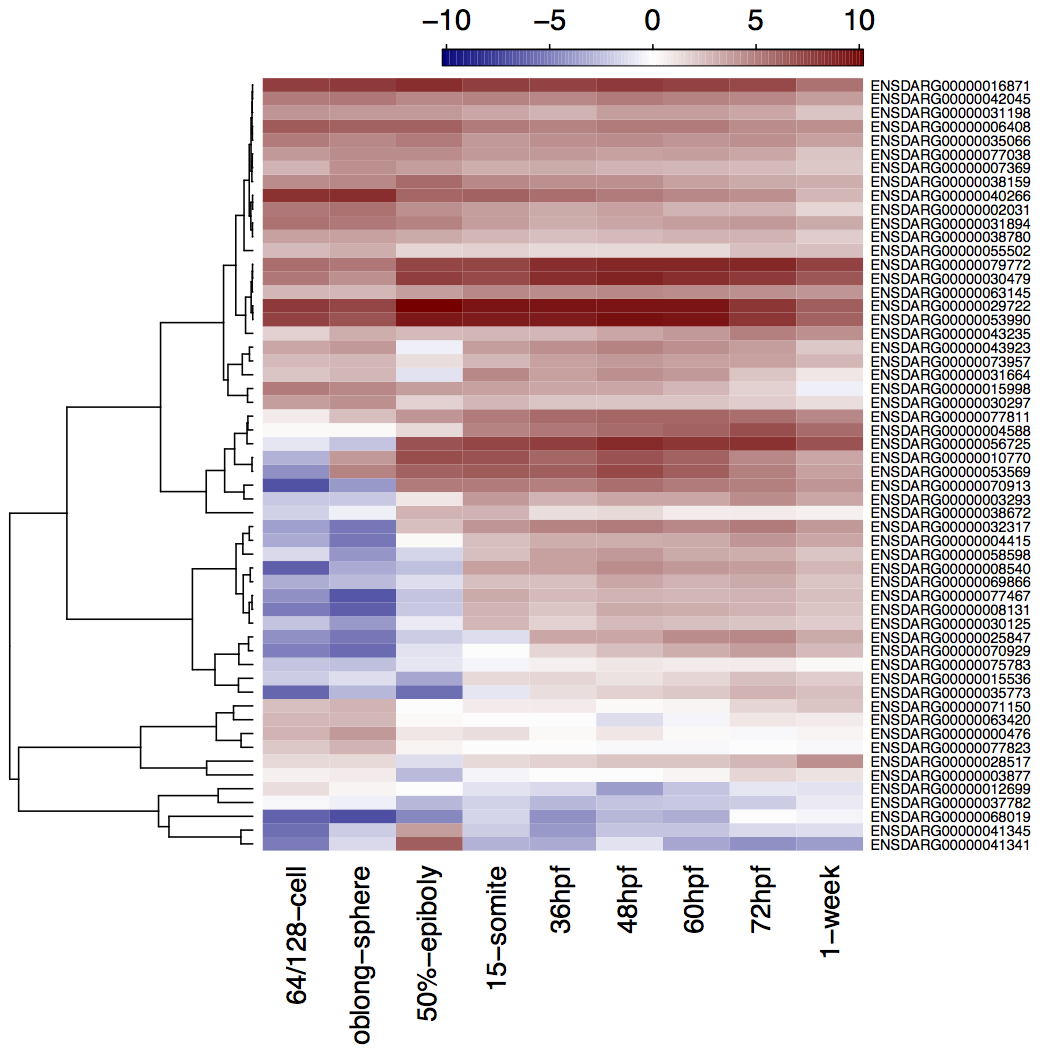

Supplement: Figure S9 — The expression of the HMG-box genes during the early development of the zebrafish. The color gradient shows the log2-transformed RPKM values for HMG-box genes at each developmental stage, as indicated by the colorbar. The dendrogram was obtained using the clustering algorithm ‘complete linkage’ based on the distances of the cosine metric. (TIFF) [file pone.0064058.s009.tiff]
